# Editorial: Nutrition and infections

**DOI:** 10.3389/fnut.2022.992095

**Published:** 2022-08-02

**Authors:** Jianhua Wu, Wei He

**Affiliations:** ^1^Leeds Institute for Data Analytics, University of Leeds, Leeds, United Kingdom; ^2^School of Dentistry, University of Leeds, Leeds, United Kingdom; ^3^Chronic Disease Research Institute, The Children's Hospital, National Clinical Research Center for Child Health, School of Public Health, School of Medicine, Zhejiang University, Hangzhou, China; ^4^Department of Nutrition and Food Hygiene, School of Public Health, School of Medicine, Zhejiang University, Hangzhou, China

**Keywords:** nutrition, infection, immune function, diet, deficiencies

Infection is one of the leading causes of death globally. Despite the economic development and the improvement of people's quality of life, the occurrence of infection is still very common. The individual dietary state has a significant impact on both host susceptibility to infectious illnesses and their outcome. According to available evidence, specific and aggregate dietary deficiencies can change a host's immune response and enhance infection susceptibility. Within this context, this Research Topic called for research on the relationship between nutritional factors and specific infection as well as mechanism of action. Nutritional factors are relatively controllable and easier to be implemented for intervention compared with drugs, surgery, and other treatment methods. As represented in this Research Topic, nutritional factors and their impact on various infections were evaluated.

For instance, Siziba et al. investigated the association of human milk oligosaccharides (HMOs) concentration and the risk of otitis media and lower and upper respiratory tract infections in 667 breastfed infants. Despite the potential benefits of neonatal immune enhancement from HMOs, the study suggests that neither prominent neutral individual HMOs (ranging from 2′-FL to LNDFHs) nor acidic human milk sialyllactoses or lactose are significantly associated with a reduced or increased risk of infections in infants up to 2 years of age.

To understand the potential benefit of anti-inflammatory short-chain fatty acids (SCFA) from dietary fiber, Ni et al. conducted mice experiment to investigate impact of dietary fiber on West Nile Virus (WNV) disease and associated survival and immune profile. Despite increased fecal SCFA acetate and changes in gut microbiota composition, dietary fiber did not affect clinical scores, leukocyte infiltration into the brain, or survival. The study concluded that fiber supplementation is not effective in WNV encephalitis.

Furthermore, Xu et al. investigated the discrepancy of gut microbiota among elite athletes and young adults with different physical activities. The matching study of 63 Chinese young adults found a non-linear association between physical activity and microbiota, which may be confounded by the dietary intake. In contrast, a separate study by Li et al. found that serum albumin was associated with disability in activities of daily living, mobility, and objective physical functioning among 2,233 Chinese older adults (≥65 years) in the Chinese Longitudinal Healthy Longevity Survey. It indicates that appropriate management of poor nutritional status through lowering serum albumin levels may contribute to maintaining physical functions in older adults.

This Research Topic includes three review articles on nutrients and infections. Govers et al. provided a broad overview on the topic of nutrition and viral respiratory tract infections. This review covered which population subgroups are susceptible to respiratory infections, which immunological mechanism contribute to antiviral immunity, how nutritional components can modulate antiviral immune response and infections. In a separate review, Cai et al. conducted a comprehensive systematic review and meta-analysis to clarify the relationship between common types of vitamins and helicobacter pylori (*H. pylori*). The study found that *H. pylori* infections decrease the serum levels of several types of vitamins, eradication of *H. pylori* could rescue its adverse effects, and antioxidant vitamin supplementation may improve the *H. pylori* eradication rate.

Furthermore, a systematic review and meta-analysis on the association between vitamin D and influenza was conducted by Zhu et al. Of the nine included randomized controlled trials, the summarized results indicates that substitution of vitamin D significantly reduces the risk of influenza infections (relative risk = 0.95, 95% confidence interval: 0.73–0.98). The study confirms the preventive effect of influenza by vitamin D supplement, but also suggests further high-quality studies with better design and larger samples.

On topics in relation to nutrient intake, Lin et al. evaluated the role of oropharyngeal dysphagia in older patients on long-term enteral feeding for risk stratification of pneumonia requiring hospitalization. It is found that the risk of pneumonia requiring hospitalization was significantly lower in patients with percutaneous endoscopic gastrostomy than in those with nasogastric tube among the patients with oropharyngeal dysphagia (adjusted hazard ratio 0.26, 95% confidence interval: 0.11–0.63). Tao et al. investigated the association between stress hyperglycaemia and stroke-associated pneumonia (SAP) with a total of 2,039 patients. The study quantified the stress hyperglycaemia through dividing the blood glucose by the glycated hemoglobin to calculate the stress hyperglycaemia ratio (SHR). It is found that SHR was significantly associated with the risk of SAP in patients without diabetes, but not in patients with diabetes. Furthermore, to predict potential surgical site infections (SSI), Chen et al. developed and validated a nomogram based on geriatric nutritional risk index (GNRI) for gynaecologic oncology patients. The use of GNRI cut-off value of 101.7 could stratify patients into distinct SSI risk groups, and the use of GNRI in nomogram model could enhance its potential to predict SSI in gynaecologic oncology patients.

Nutritional factors can support immune function against infections *via* different mechanisms and at different levels as demonstrated in this *Research Topic*. It has been shown that dietary deficiencies and malnutrition could lead to infection and contribute to morbidity and mortality. Thurs, further research should focus on identifying these nutritional factors and developing preventive strategies to target diet and nutrition at population level to improve people's quality of life.

## Author contributions

All authors listed have made a substantial, direct, and intellectual contribution to the work and approved it for publication.

## Conflict of interest

The authors declare that the research was conducted in the absence of any commercial or financial relationships that could be construed as a potential conflict of interest.

## Publisher's note

All claims expressed in this article are solely those of the authors and do not necessarily represent those of their affiliated organizations, or those of the publisher, the editors and the reviewers. Any product that may be evaluated in this article, or claim that may be made by its manufacturer, is not guaranteed or endorsed by the publisher.

